# CILIA: before and after

**DOI:** 10.1186/s13630-017-0046-8

**Published:** 2017-03-08

**Authors:** Peter Satir

**Affiliations:** Department of Anatomy and Structural Biology, Albert Einstein College of Medicine, Bronx, NY USA

**Keywords:** Ciliary motility, Primary cilia, Intraflagellar transport (IFT), Transition zone, Ciliopathies, Ciliogenesis, Nucleoporin, Autophagy

## Abstract

This is a history of cilia research before and after the discovery of intraflagellar transport (IFT) and the link between primary cilia ciliogenesis and polycystic kidney disease (PKD). Before IFT, ca. the beginning of the new millennium, although sensory and primary cilia were well described, research was largely focused on motile cilia, their structure, movement, and biogenesis. After IFT and the link to PKD, although work on motile cilia has continued to progress, research on primary cilia has exploded, leading to new insights into the role of cilia in cell signaling and development. Genomics, proteomics, and new imaging techniques have unified the field and pointed out the critical role of cilia as a restricted cell organellar compartment, functionally integrated with other cell organelles including the autophagosome and the nucleus.

## Before

Cilia are the oldest known organelle, discovered by Leewenhoek around 1674–5, because of their motility. In the era of light microscopy, motile multiciliated cells and metachronism were described, and cilia and flagella were distinguished by length, number, and beat form, but were considered “different modifications of a single type” [1]; the equivalence of basal body and centriole was postulated, single cilia (now primary cilia) were described on epithelia and a signaling function was sometimes imputed to them, and the outer segment of the vertebrate eye was thought to be derived from a single cilium. A brief history of motile cilia discovery to 1995 with key references is traced in Satir [2]; a similar history of primary cilia discovery is traced in Bloodgood [3].

Despite its usefulness, the light microscope of the 19th and early 20th century had its limitations in the study of cilia, primarily because the diameter of cilia, basal bodies, and centrioles is roughly at the limit of resolution. Non-motile cilia could be distinguished from other cell extensions in stained preparations by the most careful microscopists because of their underlying basal body, but mistakes occurred. Hence, the thickened microvilli of hair cells of the epididymis and later in the ear were labeled ‘stereocilia’ and the motility of the bacterial flagellum was compared to the flagella of eukaryotes (discussed in [4]).

In the mid-1950s, the electron microscope overcame the resolution problem and there was an explosion of studies on cilia and centrioles, at first designed to establish the basic patterns of subunits of the axoneme, basal body, and centriole. The major contributor to the electron microscopic description of cilia and cilia-related organelles was Keith Porter who in his Harvey Lecture [5] recognized that the 9 + 2 pattern was universal for motile cilia and that the axonemes of non-motile sensory ‘modified cilia’ such as the vertebrate rod or crown cells of the saccus vasculosus of fish were 9 + 0, missing the central pair, both patterns related to the nine-fold symmetry of the basal body, centriole. This was further documented in a collage of images from various investigators for motile 9 + 2 cilia of plant and mammalian sperm, 9 + 0 images of insect and invertebrate sensory cilia, several images of the connecting cilium of the developing vertebrate photoreceptor outer segment, and improved images of basal bodies and centrioles [6]. Porter and others pointed out that the axoneme was a specialized compartment of the cytoplasm enclosed in a specialized outpocketing of the cell membrane, the ciliary membrane. The term ‘microtubule’ (MT) was not in common use until 1963 [7], then leading to the now familiar descriptions; The 9 + 2 axoneme consists of 9 sets of doublet MTs surrounding a central singlet MT pair which is missing in 9 + 0 axonemes. At the distal end of the axoneme, the pattern changes, usually simplifying in the case of 9 + 2 cilia, but often with special adaptations, additional structure, or membrane adaptations in sensory cilia. Basal bodies and centrioles are composed of 9 sets of triplet MTs. From basal body to ciliary axoneme, there is a transition zone where triplet MTs become doublets [8]. Freeze fracture studies [9] later added a universal feature of the transition zone, the ciliary necklace, with an intramembrane as well as a matrix component.

Although there were sporadic reports of the presence of primary cilia in the late 1950s, in the early 1960s, 9 + 0 cilia were found to be present in a variety of ordinary mammalian tissues (mostly from rat, mouse, and chick), and on cells in tissue culture (summarized in [10]). Influential reports of this era include Barnes [11] on ultrastructure, Sorokin [12] on fibroblast primary cilia, Latta et al. [13] on kidney primary cilia, Grillo and Palay [14] on ciliated Schwann cells, and Dahl [15] on neuronal cilia. The name ‘primary cilium’ comes from a later study [16]. Because of their lack of dynein arms, 9 + 0 cilia were thought to be (and are generally) non-motile, but since no obvious function was easily observable (and cilia weren’t present in yeast), they were largely neglected and sometimes forgotten as a cell organelle. In the next decades after their discovery, people who considered them seriously proposed two hypotheses: (1) they were a way of removing the centriole from the cell cycle, particularly with entry into G_0_, thus delaying or preventing cell [17–20] and/or (2) they were sensory antennae much like the 9 + 0 sensilla of insects, capable of responding to chemical or mechanical stimuli, including flow [21–23].

In contrast to primary cilia, in the period between the electron microscope description of the cilium and the turn of the century, motile cilia were not neglected and much was learned about the structural and biochemical requirements for ciliary motility and about ciliogenesis. This was in part due to a continuous group of investigators, stemming from Sir James Gray in the Zoology Department at Cambridge University in mid-century and continuing first in Britain (Rothschild, Bradfield, Grimstone, Gibbons, Brokaw, Sleigh, Randall, Holwill etc.) and then in the United States, primarily in Chicago (Child, Satir, Rosenbaum, Borisy, Tamm, Warner, Witman etc.), and later at Rockefeller (Luck, Piperno, Huang, Dutcher etc.). To these groups were added people primarily studying sperm (Fawcett, Afzelius, Baccetti, Phillips etc.) and an important group in Japan, a tradition stemming from the discovery the ciliated sperm of *Gingko*, continuing in Tokyo via Kinoshita to Naitoh, Takahashi,Shingyoji, Kamiya etc. and elsewhere via Ogawa, Miki-Noumura etc. From a small original contingent, these groups grew large enough for the first conferences devoted to cilia to emerge. Important meetings were a Cold Spring Harbor Conference on Cell Motility, part of which (section 8, Book C) was devoted to cilia and flagella [24]; the Society for Experimental Biology meeting in Cambridge on Prokaryotic and Eukaryotic Flagella in 1980 [25]; the International Congress on Ciliary Motility and Mucociliary Transport in Friday Harbor in 1981 [26]; the International Conference on Development and Function of Cilia and Sperm Flagella in Siena in 1982 [27]; and two US–Japan joint meetings in Hakone, Japan.

From the work of these investigators, the sliding microtubule model of ciliary motility emerged, with axonemal dynein as the principal molecular motor and Ca^2+^as an important regulator of beat. These conclusions were facilitated by the developing understanding of the lipid bilayer “fluid mosaic” cell and ciliary membrane, which implied that the ciliary membrane could contain unique Ca^2+^ channels (and presumably other proteins that functioned as channels, receptors, or structural anchors) and could be removed by detergent leaving a demembranted axoneme exposed to experimental solutions. An important conclusion was that the axoneme contained all the structures and macromolecules necessary for ciliary motility and behavioral response to second messengers, so that beat could be reactivated by addition of ATP in simple solutions of appropriate pH and ion composition and beat form or frequency could be altered by the addition of second messengers such as Ca^2+^ and cAMP. Many advances were due to studies of sea urchin sperm, molluscan gills, mammalian tracheal epithelium, and ciliates such as *Tetrahymena* or *Paramecium*, while *Chlamydomonas* mutants emerged as a tool for dissecting structure and ciliogenesis. Two key questions that puzzled the field since the first observations of cilia were settled: (1) 9 + 2 motile cilia and eukaryotic flagella were virtually identical organelles with the same basic biochemical mechanisms of motion generation and control, even where motion phenotype (flexural with a clear effective stroke vs. undulatory), number, and length were quite different, and (2) metachronism was based on hydrodynamic interactions, not a membrane-based “neuroidal” mechanism. Cell coupling via gap junctions facilitated behavior such as spreading ciliary arrest, but was not necessary for metachronal activity.

As genetic and biochemical tools became more powerful, the complexity of ciliary motion became clearer. The availability of temperature-sensitive and suppressor mutants [28] was key in early proteomics of axonemal structure [29].

Axonemal dyneins, part of a larger family of dynein molecular motors, were cloned and dissected. The inner and outer dynein arms were shown to be different in regards to isoforms and arrangements of axonemal dynein, in position of the isoforms along the fundamental 96-nm periodicity of axonemal structure and in control of beat generation. The outer arm dyneins, largely uniform in composition, were shown to be minus-end MT motors, and assays pertaining to the duty cycle and mechanochemistry were developed. One implication of these assays was that dynein activity states vary during a beat, essentially switching from active to inactive. The switch point hypothesis postulates that during part of the beat, say principal bend formation, dyneins on one set of doublet MTs are active, while an opposing set are inactive, and this reverses for an opposing part of the beat (recovery bend formation) [30].

Study of motile cilia also produced the first ciliopathy description. Afzelius [31] examined the immotile spermatozoa of patients with Kartageners syndrome by electron microscopy and showed that the outer dynein arms were missing from the axoneme. He then re-analyzed other symptoms associated with the syndrome and concluded that the dynein arm ciliary motility defect in different tissues gave rise to all the symptoms: male infertility, bronchiectasis, probably hydrocephalus, and—here was a difficulty—situs inversus totalis. The difficulty was that no one had shown that there were cilia on cells at the embryonic node, the site of gastrulation thought to determine left–right asymmetry.

By 1990, the cilia field seemed to have reached a state of maturity, with incremental advances in study with each passing year. This was illusory—coupled to major advances in microscopy, biochemistry, development of genetic technologies and so on, an explosion was being ignited. One advance was the development of the air/liquid interface culture to study ciliary transport in mammalian respiratory epithelium. A second was the recognition that since cilia grew from the tip and there was no synthetic machinery within the cilium proper, ciliogenesis required transport of materials from the cell. This intraciliary/intraflagellar transport (IFT) was first described in *Chlamydamonas* motile cilia (traditionally called flagella) in Rosenbaum’s laboratory by Kozminski et al. [32, 33], then in *Caenorhabditis elegans* sensory cilia, and vertebrate photoreceptors. These and other developments by the end of the 1990s were discussed in an ad hoc international meeting on Cilia, Mucus, and Mucociliary Interaction in 1997 [34] which evolved into a continuing Gordon Conference.

## Ignition

By 1998, IFT proteins present in two complexes (A and B) had been identified [35, 36] and the IFT machinery was known to be orthologous in building cilia of many different organisms. Kinesin II was shown to be the general anterograde IFT motor, while the retrograde motor was the cytoplasmic dynein 2 (dynein 1b). In Japan, with improvements in imaging and physiological measurements, Hirokawa and associates [37] were able to show that murine embryonic node cells did indeed possess cilia and that these were a special class of motile primary cilia with dynein arms. Left–right asymmetry defects were associated with the loss of the cilia after knockdown of a component of Kinesin II, the anterograde IFT motor necessary for ciliogenesis. Motile nodal cilia were needed to produce fluid flow toward the left side of the node.

At the turn of the century, the discovery of IFT coincided with the new advances in molecular genetics that gave rise to transgenic mice and to easy Blast searches of gene and protein databases. A transgenic mouse for Autosomal Recessive Polycystic Kidney Disease (ARPKD) was developed at Oak Ridge in 1994 [38]. The gene, here called *Tg737*, produced an unknown protein, later named polaris. One allele, *Tg737* ^*orpk*^, is a hypomorphic allele and produces mice that survive until birth, while another allele is a knockout allele where animals die in mid-gestation with left–right asymmetry defects [39].

When the sequencing and cloning of *Chlamydomonas* IFT genes was begun, Pazour et al. [40] chose the IFT88 gene to sequence. A Blast search of mammalian databases revealed that the Chlamydomonas gene was orthologous to mouse *Tg737*, so that in retrospect, ‘polaris’ was actually IFT88, necessary to build primary cilia of the kidney. The hypomorphic mutant of the gene *Tg737* ^*orpk*^ produced less useful protein, leading to shorter cilia on fewer cells, as could be seen with Scanning Electron Microscopy of the kidney. The knockout allele with no cilia was more detrimental. Presumably, a similar lack of cilia or their poorer function led to ARPKD.

Praetorius and Spring [41, 42] provided a rationale for the link between the cilium and the disease. They studied fluid flow over kidney cells (MDCK) in tissue culture. Flow bent the cilia and led to an influx of intracellular Ca^2+^, initiating a signal cascade presumably necessary for normal cell replacement in the kidney. Whether this is the sole or most important effect of flow that affects cyst production is still controversial and if so, exactly how Ca^2+^ acts to prevent cyst formation is still unknown.

Notwithstanding this critical link between cilia and cystic kidneys, the major proteins mutated in polycystic kidney disease (PKD), polycystins 1 and 2 (PC1, PC2), seemed to have nothing to do with cilia. They were known to be localized in ER—but of course this was at the time that many investigators did not realize that kidney cells had primary cilia—so initially the cilia hypothesis was highly controversial. However, in *C. elegans*, Barr and colleagues [43, 44] found that homologs of PC1 and PC2 were found in sensory cilia. Both PC1 and PC2 are transmembrane proteins; PC2 is a TRP Ca^2+^ channel—that is exactly the type of channel that might respond to flow. All transmembrane proteins move through the ER on their way to the cell or ciliary membrane; so, it seemed possible that PC1 and PC2 functioned together as a flow sensor specifically in the ciliary membrane. Pazour et al. [45] and Yoder et al. [46] showed that this was likely to be the case.

In short order, the links between ciliary proteins and pathology expanded. Nauli et al. [47] and Nauli and Zhou [48] used another transgenic mouse (*Pkd1* ^−)^ defective in PC1 to show that PC1 and PC2 functioned together in the kidney cilium in that in homozygotes (*Pkd1* ^−*/*−^) while cilia were produced, neither PC1 nor PC2 actually entered cilium and the mice developed PKD. In heterozygotes, both proteins were localized to cilia and no pathology developed.

Ansley et al. [49] found that BBS proteins involved in Bardet-Biedl syndrome causing pleiotropic effects including obesity, retinal degeneration, kidney malformations, polydactyly, and brain disorders were localized to basal bodies and cilia. A more complete understanding of the comparative genomics [50, 51] and of the proteomics [52, 53] of cilia made the connection between these effects clearer. These studies suggested that a significant portion, perhaps 10%, of the human genome was devoted to cilia and ciliogenesis. Pathologies of various kinds such as PKD and Bardet-Biedl disease and variations such as Joubert or Meckel syndromes caused by ciliary malfunction began to be classified as ciliopathies.

Major signaling systems necessary for human development were found to require ciliary localization of key receptor and signaling molecules. Huangfu and Anderson [54] concluded that hedgehog signaling in the mouse ran through primary cilia, and Schneider et al. [55] showed that in fibroblast, PDGFRαα signaling was initiated in the primary cilium.

## After

These discoveries focused the attention of many laboratories and produced an explosion of new investigators working on both motile and primary cilia, leading to several new volumes of collected papers, notably “Ciliary function in mammalian development” edited by Bradley Yoder [56], a series for methods in cell biology edited by Stephen King and Gregory Pazour [57–59] and Roger Sloboda [60], a small symposium in 2010 on “The New Biology of Cilia” [61], and new meeting series beginning in 2012 including the continuing FASEB SRC on “The Biology of Cilia and Flagella,” a Keystone Symposium and a continuing EMBO/International Cilia Conference in Europe. By 2012, the number of publications on cilia was such that a new journal—*Cilia*—was founded to facilitate the timely publication of “a wide range of topics from the structure of cilia to human genetics to ciliotherapeutics” [62] in an open access journal. Readers of *Cilia* know that each volume contains a wealth of new information and important reviews of the field. It would be presumptuous and far beyond the scope of this article to provide a comprehensive review of the subject or the literature since the beginning of this era. Instead, this section will highlight a few selected studies that are of special interest to me and which might illuminate certain future directions of the subject.

### Cryo-electron microscope tomography of motile cilia

Just as thin-section transmission electron microscopy initiated a new era in the study of ciliary structure, leading to the switch point hypothesis as described above, cryo-EM tomography has opened the possibility of understanding the structural changes that accompany beat at a molecular level. Cryo-EM of cilia has been reviewed by Ishikawa [63]. Although cilia of different organisms look similar, it is surprising (but perhaps not entirely unexpected) that this similarity persists at high cryo-EM resolution [64, 65], that is at the most intimate molecular structural level, and differences can be utilized in clinical diagnosis of ciliopathies [66]. New features such as the identification of the dynein regulatory complex (DRC) with the (nexin) interdoublet link [67] fit biochemical and structural information together.

Importantly, dynein stroke stages seen in isolated cytoplasmic dynein can be discerned in complex three-headed outer dynein arms along beating cilia or sperm tails [63, 68]. Spoke structure and attachment to the central sheath has also been examined with cryo-EM. [69–73] but spoke–central sheath attachment has not yet been constructed simultaneously for all doublets in bent vs straight portions of the axoneme nor has this been correlated with dynein stroke stages of the same doublets. In other words, although tremendous progress has been made, we still do not know the answer to the fundamental question of how a bend forms and progresses to produce ciliary beat. This is the question I wished to address when I began my work over 50 years ago. We now seem to have the tools to answer it.

### CLEM of IFT

The possibility of capturing structural signatures of dynamic events such as bend formation during a ciliary beat might seem far fetched, but an expansion of cryo-electron microscopy by CLEM, correlative light and electron microscopy, may make this possible. The technique has been pioneered in a brilliant study of IFT [74]. The dynamic feature being studied—in this case, anterograde and retrograde IFT—is recorded and fast fixed at a given timepoint for microscopy. In this case, IFT trains were seen in TIRF microscopy and fluorescent color coding was added after fixation to indicate which particular particles moved anterograde vs retrograde, but in other cases, fluorescent markers of, for example, a particular protein could be part of the original specimen. Then the same specimen was prepared for cryo-electron microscope tomography and the light and EM images overlaid by CLEM. The known positions of anterograde and retrograde IFT trains could then be found and studied at high resolution. The conclusion that anterograde IFT moves along the B subtubule of the doublet while retrograde IFT moves along the A subtubule would be difficult to demonstrate without this correlative technique. One can readily imagine extensions of this study to examine other features of IFT, including cargo traffic, tip exchange, and train assembly, but the technique should be capable of much wider application to dynamic cell processes, for example capturing bend progression in the fixed metachronal wave, fulfilling my original dream.

### Superresolution of the transition zone—relation to composition of the ciliary necklace

Protein entry into the cilium is highly selective; for example, in fibroblasts under physiological conditions, the transmembrane protein PDGFRα (a receptor tyrosine kinase) enters the cilium and functions exclusively therein, while the related receptor PDGFRβ never enters [55]. Likewise, in mammalian cells, certain G-protein-coupled receptors (GPCRs) must enter the cilium in order to function appropriately [75, 76]. The barrier to entry and its selectivity appears to lie at the base of the cilium, the transition zone where a ciliary pore structure is nearly universally present. This is the structure originally described as the ciliary necklace (see Before) because of the appearance of rows of intramembrane particles (IMPs), connected by the pores to the doublet microtubules (the so-called Y-links),. The molecular composition and arrangement of the transition zone has taken on special importance because these are the molecules which when mutated give rise to syndromic ciliopathies such as Joubert, Meckel-Gruber, Bardet-Biedel syndromes, and other human pathologies. The nephronophthisis (NPHP) family of proteins are mutated in many of these pathologies and together with the Meckel syndrome (MKS) proteins perhaps hold the key to barrier construction and function. Some small cytosolic proteins enter the cilium by diffusion, although there is some controversy about the exact size of the diffusion barrier. However, it seems likely that very large cytosolic molecules such as dynein complexes, IFT complexes and both peripheral and especially transmembrane proteins require special mechanisms of entry, including interaction with the ciliary pores. A major player in forming the matrix pore-membrane Y-link connections through which large cytoplasmic complexes pass is NPHP6, also called CEP 290. This protein has an interacting partner CC2D2A (MKS6). The NPHP6/CC2D2A heterodimer also interacts with other members of the NPHP family including NPHP1, NPHP4, and with RPGRIP1L (NPHP8) [77, 78]. Peripheral and trans- membrane proteins must pass through the barrier represented by the necklace IMPs, whose likely components include two transmembrane proteins TMEM67and TMEM231 and the tectonic proteins, TCTN1 and TCTN2

**Fig. 1:**
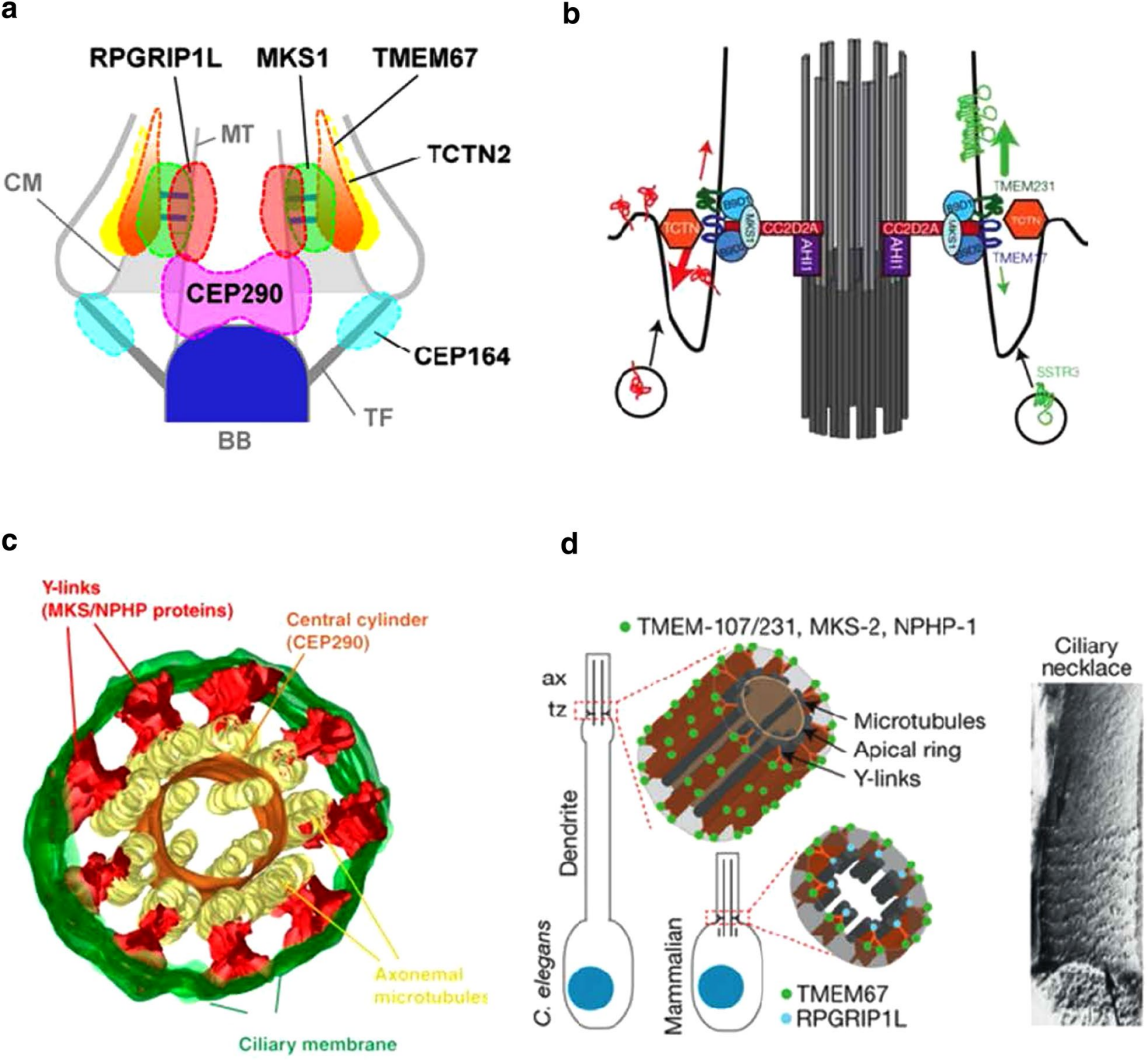
Current models of protein localization in the transition zone. **a** Localization based on aligned superresolution images of multiple single-colored STED images and an EM image of a RPE-1 cell primary cilium. TMEM67 and TCTN2 lie at the ciliary membrane in the ciliary necklace region. MSK1 is in the area of the ciliary pores (Y-links), while RPGRIP1L is at the same level but more proximal to the axonemal MTs; CEP290 is at the base of the zone. With permission from [79], courtesy of Jung-Chi Laio. **b** The position of the B9D1 complex proteins in relation to other transition zone proteins. In IMCD cells growing primary cilia, TCTN, TMEM 231, and TMEM17 lie in positions to be part of the ciliary necklace. An MSK1, B9D1-2 complex is anchored to the ciliary membrane at the level of the ciliary pores, and the stem of the Y-link is formed by CC2D2A resting on Jouberin (AHI-1). The positions of CEP290 and RPGRIP1L are not specified. With permission from [81], courtesy of Andrew S. Peterson. **c** Protein localization in the transition zone of *C. elegans* based on video reconstruction using mutant phenotypes. CEP-290 is a component of the apical ring, a central cylinder internal to the axonemal MTs and MKS and NPHP modules form the ciliary pores/Y-links. With permission from [82], courtesy of Alexander Dammermann. **d** A second model of the *C. elegans* transition zone based on protein localization, FRAP, and superresolution microscopy. RPGRIP1L and TMEM67 lie at the base of the zone forming a ring extending from the axoneme to the membrane. Distally MKS and NPHP proteins form rings or spirals whose periodicity and immobility correspond to the Y-links and ciliary necklace. From [83], courtesy of Oliver E. Blacque

Superresolution microscopy in conjunction with TEM CLEM has begun to reveal positional information with respect to the ciliary pores. Yang et al. [79] have produced a map of the positions of important proteins. Y-links are at the level where RPGRIP1L and MKS1 are localized, and TMEM67 and TCTN2 are perhaps the ciliary necklace IMPS (Fig. 1a). CEP290, which others identify as part of the Y-links in *Chlamydomonas* [80], is at a more basal level. To form the barrier for membrane proteins, many of these proteins may interact at the transition zone with the B9 protein complex protein B9D1 [81] (Fig. 1b).

*Caenorhabditis elegans* provides a slightly different picture [82] with CEP290 forming a central cylinder and MKS and NPHP proteins forming the Y-links extending from the cylinder (Fig. 1c). A key protein is TMEM107 which functions with NPHP4. TMEM107 organizes recruitment of MKS1, TMEM231 (JBTS20) and TMEM237 (JBTS14), and MKS module membrane proteins in rings or spirals corresponding to the ciliary necklace and Y-links above a basal ring comprised of TMEM67 and RPGRIP 1L [83] (Fig. 1d). A model for sequential assembly for the *C. elegans* transition zone based on MKS5 and CEP290 has been proposed [84]. It seems probable that when differences between phyla are sorted out, the structural organization of the transition zone will prove to be highly conserved and related to the selective mechanism for sorting molecules into and out of the cilium

In this regard, the ciliary pore/Y-links separate the A and B subtubules of each doublet. This suggests that IFTB complexes entering the cilium move along one side of the pore (one branch of the Y) separated from IFTA complexes leaving the cilium on the opposite side. Presumably IFT particles become fully assembled or disassembled as they pass through the transition zone.

They must be attached to or dissociated from molecular motors and cargo, including transmembrane proteins before or just after leaving the zone.

These complex assembly/disassembly processes present many unsolved questions. They all occur in a limited space and time, both requiring further delineation, which we surely will see future work.

### Evolution, import signals, and nucleoporins

Because of common evolutionary origin of all cilia, functional organization of the transition zone, that is transport through the transition zone/pore/necklace barrier, assembly and co-transport with IFT complexes and molecular motors should also be largely conserved among cilia of different organisms. This should hold true both for cytosolic proteins diffusing or actively transported into the axonemal matrix space (cilioplasm) and for transport of both peripheral membrane scaffold proteins and transmembrane proteins.

The motile 9 + 2 cilium arose early in eukaryotic evolution and was probably present in LECA, the last common ancestor of all eukaryotes. One suggestion is that the cilium arose by viral invasion in the same cell lineage as the eukaryotic nucleus, and more particularly the nuclear membrane, was evolving and that LECA had both these functioning organelles: the cilium for the advantage of efficient motility and the nucleus for the advantage of efficient information transfer and chromosomal replication and distribution. In this case, the barriers of the cilium and the nucleus probably evolved together from scaffold proteins that were becoming structures such as coat protein complexes (COPs) and nucleoporins. This might imply that the ciliary necklace pores and nuclear pores have similar protein constituents and mechanisms of molecular trafficking [85]. Orthologous molecules include Ran-GTP [86], RCC1, Importin and export factors [87], and probably nucleoporins. In *Xenopus,* inner ring nucleoporins (nup 188, nup 93) localize to the ciliary base. Depletion or overexpression of nup 188 affects ciliogenesis and left–right asymmetry, leading to heart defects [88]. The cilioplasm and the nucleoplasm would have arisen as privileged compartments with protein selectivity via a pore barrier defining their contents and signaling resulting in specific gene activation as selected proteins moved from cilium to nucleus [89, 90].

What is not in question is that many cytoplasmic proteins, including proteins that carry signals from ciliary receptors, are selectively or exclusively found in the cilium at some time in the cell cycle and that under certain conditions, these proteins move readily from cilium to nucleus to affect gene expression. The classic example is Gli [91] which is localized to the cilium tip, but during hedgehog signaling is specially processed and leaves the cilium to enter the nucleus to affect transcription. Many other signaling molecules including Smad transcription factors [92], p90Rsk [93], Jade 1, a Wnt-related ubiquitin ligase [94], huntingtin [87], parafusin [90], pVHL [95, 96], and RSP3 [97] move from the cilium to the nucleus and sometimes vice versa, meaning that they are recognized and moved past both the ciliary and nuclear barriers. How transport through the cytoplasm between the organelles occurs is unknown—perhaps by diffusion, perhaps requiring cytoskeletal elements and molecular motors. Similar nucleoporins apparently line both the ciliary and nuclear pores [98]. Selective shuttling past similarly constructed barriers suggests that the mechanisms of import and export are similar and homologous, but probably not quite identical for the two organelles. This has now been demonstrated for Gli2 [99] and may apply generally to cytosolic proteins, including ciliary motor molecules such as Kif17 [89].

However, while only one traverse of the nuclear pore is required for entry or exit, the ciliary transition zone consists of reiterating rows of pores, each row also defined by its row of necklace IMPs. Cilia of some organisms (e.g., *Chlamydomonas*) have as few as two rows, but the connecting cilium of mammalian photoreceptors may have more than 25 rows. It seems likely that the number of rows is related to a recurrent transport mechanism within the transition zone. Especially for peripheral and transmembrane proteins, import and export selectivity might increase at each ciliary pore (Y-link) and at each necklace row. Although certain molecules—e.g., the BBsome proteins and TULP3 [100], and the NPHP and MKS modules and organization (Fig. 1) discussed in the previous section– are known to be implicated in this mechanism, a detailed understanding is still elusive.

Cilia of certain plants are missing the Y-link ciliary pores and their constituent proteins entirely [101]. Plant cilia are found on gametes, which, for both plants and animals, may have different transport requirements and selectivity mechanisms from somatic cilia, permitting divergent evolution.

It would be important to see a definitive model of transport through the ciliary transition zone developed in molecular detail; it would also be interesting to learn the details of transport between nucleus and cilium. Will there be further evidence suggesting that the hypothesis of common origin of the two organelles is correct?

### Integration with autophagy

Although the cilium at any one time contains a specially selected set of cytoskeletal, matrix and membrane molecules, it cannot be considered to be isolated from other cell processes, because it is in constant communication with the rest of the cell, not only by molecular trafficking to the nucleus but also to other aspects of cellular metabolism. There is a clear reciprocal relationship between ciliogenesis and autophagy [102, 103]. Basal autophagy in MEFs cultured in serum inhibits ciliary growth in part because proteins such as IFT20, transported to the cilium in Golgi-derived vesicles, are degraded in the autophagosome and unavailable for efficient ciliogenesis. Where autophagy is deficient, ciliogenesis and cilia length increase. Upon serum starvation, ciliogenesis is induced and autophagy increases with roughly the same time course. Early upon serum removal, when autophagy is also activated, cilia growth is possible because autophagy degrades OFD1, a ciliogenesis inhibitor [96]. Atg16L, a component of early autophagosome formation, is transported from the Golgi together with IFT20, and as it becomes localized along the cilium or at its base, could trigger the increase in plasma membrane or ciliary pocket-associated autophagy. Ciliary signaling pathways, particularly Hh signaling, may play a critical role in this process. Via this interaction with autophagosome formation and nutrient sensing, the cilium is interconnected to important membrane trafficking and metabolic pathways in the cell. A major link is provided by the Lkb1, AMPK, and mTOR signaling pathway which is activated by ciliary bending [104] or by flow and in turn induces autophagy which regulates cell volume [105, 106]. The ubiquitin-proteosome system may also come into play [107].

The cilium–autophagy axis has been shown to be important in hESC differentiation toward neurectoderm (NE) [108]. By day 2, increased ciliation in NE presursors increases autophagy which in turn suppresses the major transcription factor Nrf2 which guides lineage fate expression.

The relationship between cilia and major synthetic and degradative pathways of the cell (which was not entirely anticipated, but is obviously part of the integrated cell) is an exciting area for future research with significant bearing on human health and disease, including cancer [109].

### The cilium as a secretory organelle

The shedding of membrane or membrane-bound vesicles from the tips of ordinary cilia has been considered a possibility since the discovery by Young and Bok [110] of shedding of the photoreceptor outer segment and engulfment of the shedded discs by retinal pigment epithelium. Further, it is suggested by studies of protistan mating, showing that gamones are present on the ciliary membrane which could be shed into the medium. There are many cases of vesicles that are attached to and may be budding from cilia, but a major problem is whether they are coming or going. As with IFT, the best understanding of and insight into the process awaited work on *Chlamydomonas* [111]. In *Chlamydomonas*, new daughter cells are encased in their own cell walls. The cilia become surrounded by small vesicles, ciliary ectosomes which (since the only source of ectosomal membrane is the ciliary membrane) must therefore be produced from the ciliary membrane. Further work has shown that the ectosomes are enriched in a unique subset of ciliary membrane proteins, including proteins from the endosomal sorting complex required for transport (ESCRT) that may mediate ectosome release [112]. TEM images show the ectosomes budding from the cilium tips and possibly elsewhere along the cilium. The outer surface of the ectosomal membranes contains a specific protease VLE that is functionally necessary to digest the cell wall in which the new daughter cells are trapped. This demonstrates that the ectosomes have an important extracellular physiological function and establishes the process of ectosomal secretion as important for cell survival.

This work has been complemented by examination of other cases of ciliary secretion, summarized by Wood and Rosenbaum [111]. There is good evidence of vesicular secretion from *C. elegans* cilia and suggestive evidence from several other systems including mammalian primary cilia in renal and neuro-epithelium. The ectosomes may be used for cellular communication or perhaps in the case of *C. elegans* for communication between organisms. The field is ripe for intensive study and growth, with many important questions still unanswered [113].

## Concluding remarks

When the electron microscope first revealed the beautiful and consistent 9 + 2 pattern of motile cilia and the ultrastructural relationship of motile cilia to centrioles, basal bodies, and sensory (and then primary) cilia, few people could envision where the study of cilia might lead a half century later. For those of us who have remained with the field through these many years, it is a source of deep satisfaction that the study of cilia has led to so many unexpected and fundamental discoveries in cell biology, some of which have been recounted in this article. For those now entering the field, the view is much broader but no less exciting. Technological, genetic, and imaging advances have made examination of the molecular dynamics of the cilium accessible for new discoveries. The ciliopathies have introduced the field into human health and molecular medicine. Many cancers and certain intellectual and cognitive disorders have a ciliopathy component, suggesting that studies of cilia in health and disease will be of increased importance in future explorations and that understanding the cell biology of cilia may lead to new therapeutics.

## Abbreviations

IFT: intraflagellar transport
PKD: polycystic kidney disease
MT: microtubule
DRC: dynein regulatory complex
ARPKD: autosomal recessive polycystic kidney disease
MDCK: Madin-Darby canine kidney epithelial cells
PC: polycystin
ER: endoplasmic reticulum
TRP: transient receptor potential
BBS: Bardet-Biedl syndrome
PDGF: platelet derived growth factor
EM: electron microscope
TEM: transmission electron microscopy
CLEM: correlated light and electron microscopy
TIRF: total internal reflection fluorescence microscopy
LECA: last evolutionary common ancestor
COP: coat protein
GPCR: G-protein-coupled receptor
IMP: intramembrane particle
NPHP: nephronophthisis
MKS: Meckel syndrome
CEP290: centrosomal protein of 290 kDa
JBTS: Joubert syndrome
RPGRIP1L: retinitis pigmentosa GTPase regulator interacting protein 1-like
TMEM: transmembrane protein
TCTN: tectonic protein
RCC1: regulator of chromosome condensation 1
VHL: von Hippel-Lindau
RSP: radial spoke protein
TULP: tubby-like protein
OFD1: Oral-facial-digital syndrome type I
MEF: mouse embryonic fibroblast
Lkb1: liver kinase B1 (serine threonine kinase 11)
mTOR: mechanistic target of rapamycin
AMPK: adenosine monophosphate kinase
hESC: human embryonic stem cell
NE: neurectoderm
ESCRT: endosomal sorting complex required for transport
